# Phase-based treatment versus immediate trauma-focused treatment in patients with childhood trauma-related posttraumatic stress disorder: study protocol for a randomized controlled trial

**DOI:** 10.1186/s13063-018-2508-8

**Published:** 2018-02-22

**Authors:** Noortje I. van Vliet, Rafaele J. C. Huntjens, Maarten K. van Dijk, Ad de Jongh

**Affiliations:** 1 Department for Anxiety and Mood Disorders, Dimence Mental Health Group, Deventer, the Netherlands; 2 Department of Experimental Psychotherapy and Psychopathology, University of Groningen, Groningen, the Netherlands; 3 Department of Social Dentistry and Behavioral Sciences, University of Amsterdam and Vrije Universiteit, Amsterdam, the Netherlands; 4 School of Health Sciences, Salford University, Manchester, UK; 5 Institute of Health and Society, University of Worcester, Worcester, UK

**Keywords:** PTSD, Complex PTSD, Trauma-focused therapy, Treatment, STAIR, EMDR

## Abstract

**Background:**

The treatment of posttraumatic stress disorder (PTSD) related to a history of sexual and/or physical abuse in childhood is the subject of international debate, with some favouring a phase-based approach as their preferred treatment, while others argue for immediate trauma-focused treatment. A history of (chronic) traumatisation during childhood has been linked to the development of distinct symptoms that are often labelled as symptoms of complex PTSD. Many therapists associate the presence of symptoms of complex PTSD with a less favourable treatment prognosis. The purpose of this study is to determine whether a phase-based approach is more effective than stand-alone trauma-focused therapy in individuals with PTSD and possible symptoms of complex PTSD resulting from a history of repeated sexual and/or physical abuse in childhood. An additional aim is to investigate moderators, predictors of treatment (non) response and drop-out.

**Method:**

The sample consists of patients between 18 and 65 years old with a diagnosis of PTSD who report a history of repeated sexual and/or physical abuse in childhood (*N* = 122). Patients will be blindly allocated to either 16 sessions of eye movement desensitization and reprocessing (EMDR) therapy preceded by a stabilization phase (eight sessions of Skills Training in Affect and Interpersonal Regulation (STAIR)) or only 16 sessions of EMDR therapy. Assessments are carried out pre-treatment, after every eighth session, post-treatment, and at 3 and 6 months follow up. The main parameter will be the severity of PTSD symptoms (PTSD Symptoms Scale-Self Report). Secondary outcome variables are the presence of a PTSD diagnosis (Clinician-Administered PTSD Scale for DSM-5), severity of complex PTSD symptoms (Structured Interview for Disorders of Extreme Stress-Revised and symptoms-specific questionnaires), changes in symptoms of general psychopathology (Brief Symptom Inventory), and quality of life (Euroqol-5D). Health care consumption and productivity loss in patients will also be indexed.

**Discussion:**

The study results may help to inform the ongoing debate about whether a phase-based approach has added value over immediate trauma-focused therapy in patients suffering from PTSD due to childhood abuse. Furthermore, the results will contribute to knowledge about the safety, efficacy, and cost-effectiveness of treatments in this target group.

**Trial registration:**

Nederlands Trialregister, NTR5991. Registered on 23 august 2016. http://www.trialregister.nl/trialreg/admin/rctview.asp?TC=5991

**Electronic supplementary material:**

The online version of this article (10.1186/s13063-018-2508-8) contains supplementary material, which is available to authorized users.

## Background

For posttraumatic stress disorder (PTSD), international expert consensus guidelines recommend the use of evidence-based, trauma-focused psychological treatments, particularly cognitive behavioural therapy (i.e., prolonged exposure) and eye movement desensitization and reprocessing (EMDR) therapy [1, 2]. For PTSD following childhood abuse, the application of trauma-focused treatments is generally associated with reductions in PTSD symptom severity and comorbid symptoms of depression, anxiety, and dissociation [3–7].

Childhood physical and/or sexual abuse is considered to be one of the traumatic stressors associated with the development of complex PTSD [8]. The term complex PTSD was first launched by Herman [9] and is assumed to develop following the experience of severe, prolonged, and/or repeated interpersonal trauma. Complex PTSD is diagnosed when, in addition to meeting criteria for PTSD, trauma survivors exhibit disturbances in three domains of self-organization - affect regulation, self-concept, and interpersonal functioning [10] - and is currently being considered for inclusion as a separate diagnostic entity in *International Classification of Diseases 11* (ICD-11) [11].

It has been argued that individuals with a history of childhood abuse who suffer from symptoms of complex PTSD may be insufficiently stable to tolerate evidence-based immediate trauma-focused treatment [8, 12]. In 2011 the International Society for Traumatic Stress Studies (ISTSS) Complex Trauma Task Force published the results of a survey meant to obtain expert opinions about the treatment of patients with symptoms of complex PTSD [13] showing that 85% of the experts reported that they would use a phase-based approach as their first line of treatment. Based upon these results, a phase-based treatment has been recommended for these patients [8], in which trauma-focused treatment (phase II) is preceded by a stabilization phase (phase I) aimed at ensuring the individual’s safety, reducing self-regulatory problems, and improving emotional, social, and other psychological skills. This phase-based approach is then completed with a reintegration phase (phase III), aimed at consolidating treatment gains and helping the person to adapt to his or her present life circumstances [8]. In clinical practice, the phase I treatment for PTSD with symptoms of complex PTSD varies from eight weekly sessions (i.e., the programme Skills Training in Affective and Interpersonal Regulation (STAIR)) [14] up to programme with a much longer duration [15, 16]. In the guidelines of the ISTSS the majority of the experts considered 6 months as a reasonable length for phase I [8].

Until now, the safety and efficacy of the phase-based treatment approach for treating complex PTSD has been addressed in two studies [12, 14]. The first study used a randomized controlled trial (RCT) to compare the efficacy of STAIR (a phase I treatment) followed by prolonged exposure versus a waiting-list condition in a sample of female patients that suffer from PTSD as a result of childhood physical and/or sexual abuse [14]. The STAIR/exposure condition resulted in significant symptom reductions (i.e., PTSD severity, depression, general anxiety, dissociation), plus significant improvements in mood and anger regulation skills. In the STAIR phase, depression, anxiety, anger expression, and negative mood regulation improved significantly. There were no improvements in PTSD symptoms, dissociation, and alexithymia. The prolonged Exposure (PE) phase showed reductions in PTSD symptoms, dissociation, and alexithymia, and further improvements in depression and anxiety. No improvements were found in negative mood regulation and in anger expression in the PE phase. The results of this study suggest that the combination of STAIR/exposure is feasible and leads to a decrease in PTSD and a broad range of other symptoms associated with complex PTSD. One limitation of this study, however, was the relatively high drop-out rate in the STAIR/exposure condition (29% compared to 11% in the waiting list control group). Also, effect sizes pointed to the phase of exposure treatment conferring the bulk of the therapeutic benefits. Hence, although this study supports the effectiveness of a STAIR/exposure combination, the results do not provide univocal support for the contention that a phase-based treatment is superior to immediate trauma-focused treatment for individuals suffering from severe PTSD and symptoms of complex PTSD (see also [17]). Clearly, there was a need for direct comparison between a phase-based and an immediate trauma-focused treatment condition. Accordingly, in a second study, Cloitre and colleagues evaluated the efficacy of a phase-based treatment (STAIR/exposure) versus supportive counselling followed by prolonged exposure (support/ exposure) and versus STAIR followed by supportive counselling (STAIR/support) [12]. Women with PTSD related to childhood sexual and/or physical abuse were assigned to one of the three treatment conditions. The application of STAIR/exposure was found to be associated with greater benefits compared to the support/exposure condition in terms of self-reported reduction in PTSD symptom severity, interpersonal problems, and emotion regulation, but only at follow up (both 3 and 6 months). Immediately after treatment, all three experimental treatment conditions resulted in a substantial proportion of patients being PTSD negative (i.e., no significant differences were found between the three conditions). However, the lack of a treatment condition in which patients were directly exposed to their traumatic memories precludes drawing any firm conclusions about the relative benefits of a phase-based treatment approach over an immediate trauma-focused approach for patients suffering from PTSD related to childhood abuse (see De Jongh et al.) [18]. In other words, the core question is still whether the addition of a stabilization phase is a necessary condition for, and/or has added value over, immediate trauma-focused treatment. And if so, it is important to know which of the two treatment approaches works best for whom. The present study aims to address these questions.

## Methods/design

### Aim

The purpose of the current study “ToPrepareOrNot” (TOPRON), is to determine whether a phase-based approach is more effective than immediate trauma-focused treatment in individuals with PTSD related to repeated childhood abuse, and possible symptoms of complex PTSD. For the trauma-focused condition we used eye movement desensitization and reprocessing (EMDR) therapy, because it is one of the guideline treatments for PTSD [2] with an efficacy similar to PE. Our first hypotheses is based upon the current guidelines for the treatment of complex PTSD [8], and expert consensus about the treatment of this target population [13]; that is, that the phase-based treatment condition (i.e., STAIR/EMDR) is significantly more effective in PTSD (severity of PTSD symptoms and proportion of diagnoses lost) than the immediate trauma-focused condition (i.e., EMDR/EMDR). Our second hypothesis is that the phase-based treatment approach leads to a significantly better outcome in terms of reduced symptoms of complex PTSD and comorbid symptoms, drop-out rate, quality of life, and health care consumption than the immediate trauma-focused condition. An additional aim is to investigate potential moderators and predictors of drop-out or treatment (non) response. To this end, we hypothesized that signs of affect dysregulation and having interpersonal problems at the start of therapy would be related to worse outcome in the trauma-focused condition [19, 20]. We also expect patients with a comorbid personality disorder [21] and high level of dissociation at the start of therapy [22] to be more at risk of deterioration and drop-out in both conditions.

### Study design

**Fig. 1:**
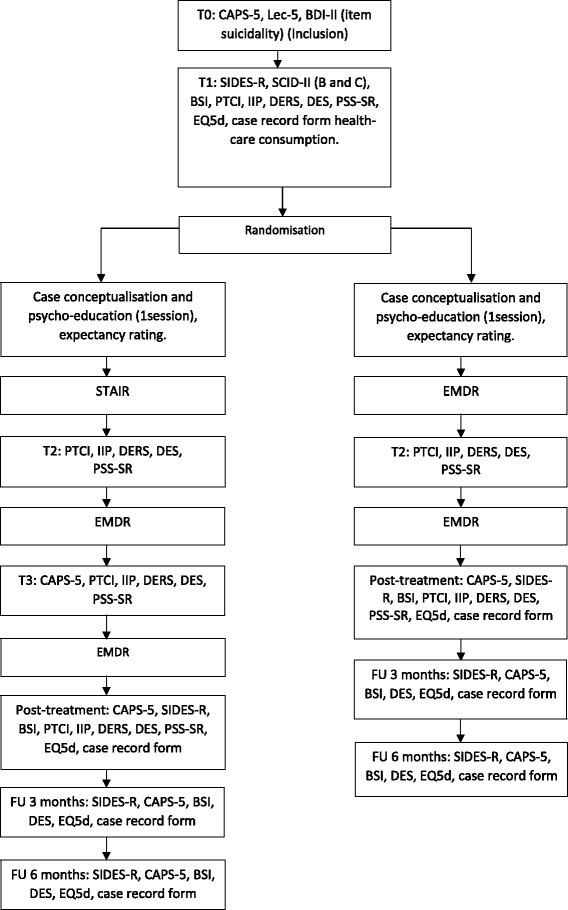
Study design, showing treatment conditions and measurements. BDI-II, Beck Depression Inventory; BSI, Brief Symptom Inventory; CAPS, Clinician-Administered PTSD Scale for DSM-5; DERS, Difficulties in Emotion Regulation Scale; DES-II, Dissociative Experiences Scale; EMDR, Eye Movement Desensitization and Reprocessing; EQ-5D, Euroqol-5D; IIP, Inventory of Interpersonal Problems; LEC-5, Life Events Checklist for DSM-5; PSS-SR, PTSD Symptoms Scale-Self Report; PTCI, Posttraumatic Cognitions Inventory; SCID-II, Structured Clinical Interview for DSM-IV Axis II; SIDES-R, Structured Interview for Disorders of Extreme Stress-Revised; STAIR, Skills Training in Affect and Interpersonal regulation; T, time point; FU, follow up

**Fig. 2:**
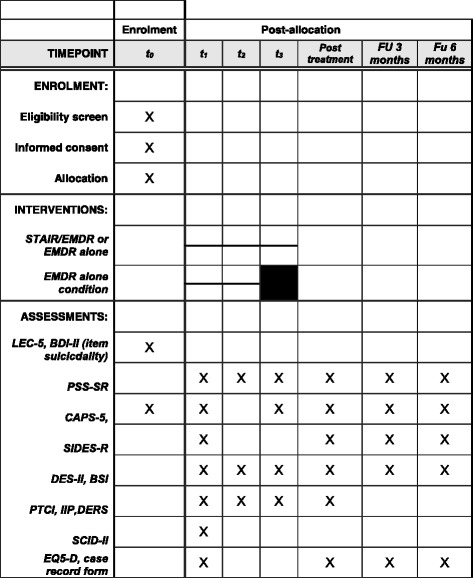
Enrolment, treatment and assessments over time. BDI-II, Beck Depression Inventory; BSI, Brief Symptom Inventory; CAPS, Clinician-Administered PTSD Scale for DSM-5; DERS, Difficulties in Emotion Regulation Scale; DES-II, Dissociative Experiences Scale; EMDR, Eye Movement Desensitization and Reprocessing; EQ-5D, Euroqol-5D; IIP, Inventory of Interpersonal Problems; LEC-5, Life Events Checklist for DSM-5; PSS-SR, PTSD Symptoms Scale-Self Report; PTCI, Posttraumatic Cognitions Inventory; SCID-II, Structured Clinical Interview for DSM-IV Axis II; SIDES-R, Structured Interview for Disorders of Extreme Stress-Revised; STAIR, Skills Training in Affect and Interpersonal regulation; *t*, time point; FU, follow up

The TOPRON study entails a randomized controlled trial with two arms: a phase-based treatment approach (STAIR-EMDR) versus a trauma-focused treatment condition (EMDR therapy only). In the STAIR-EMDR condition, patients will receive eight sessions of stabilization treatment (STAIR), followed by 16 sessions of EMDR therapy, adding up to a total number of 24 treatment sessions. In the trauma-focused condition, patients will receive 16 sessions of EMDR therapy. All sessions last 90 min. The two groups will be compared on a number of variables before treatment, after every eighth session, post-treatment and at follow up, that is 3 months and 6 months after the post-treatment session (see Figs. 1 and 2). By assessing every eight sessions it will be possible to determine the added value of a stabilization phase examining the results after an equal amount of sessions. For this study, we chose to use a fixed number of sessions. All patients in the STAIR-EMDR condition will receive eight sessions STAIR while in both conditions patients will receive a maximum of 16 sessions of EMDR. When all targets are processed to a Subjective Unit of Distress (SUD) score of 0 and a Validity of Cognition (VoC) score of 7, the patient will be assessed to determine whether he or she no longer meets the criteria for PTSD. In the case of early completion, the remaining sessions are cancelled. Because STAIR is meant as a first-phase treatment prior to the start of trauma-focused treatment (i.e., EMDR therapy) following STAIR, all patients will receive EMDR therapy. That is the reason that early completion during STAIR is not possible.

### Research setting

Patients will be recruited from various departments of Dimence, a large mental health organization in the east of the Netherlands. Dimence specialises in treating patients with severe mental illnesses.

### Participants

The inclusion criteria are (a) age between 18 and 65 years, (b) diagnosis of PTSD according to the *Diagnostic and Statistical Manual of Mental Disorders 5* (DSM-5) [23], and (c) victim of repeated sexual and/or physical abuse before the age of 18 years by a carer or a person in a position of authority (i.e., with repeated abuse defined as more than one incident by the same or different perpetrator(s)). The exclusion criteria are (a) insufficient proficiency in the Dutch language, (b) high risk of suicidality assessed by the Beck Depression Inventory (BDI)-II [24]), (c) treatment for PTSD in the past year with at least eight sessions (EMDR, prolonged exposure, or any well-evaluated stabilization programme), (d) alcohol or drug dependence or abuse according to DSM-IV-TR [25], (e) mental retardation, and (f) victim of ongoing physical and/or sexual abuse.

### Interventions

STAIR [14] will be applied In the first treatment phase (phase I, stabilization). The goals of this treatment are (a) addressing problems in affect and interpersonal regulation and (b) preparing the individual for the effective and successful use of the trauma-focused treatment [14]. The programme consists of eight sessions with different topics (introduction, emotional awareness, emotion regulation, emotionally engaged living, understanding relationship patterns, changing relationship patterns, agency in relationships, and flexibility in relationships). All STAIR sessions have more or less an identical format and structure: psycho-education about the rationale and goals of the interventions, skills acquisition, skills application, and practice. The therapeutic techniques employed include for example identifying and exploring cognitive schemes, role-playing, behavioural exercises at home, and respiration exercises.

The trauma-focused element in therapy will be EMDR therapy, which is a protocolised evidence-based trauma treatment, aimed at resolving symptoms as a result of disturbing or unprocessed life experiences [26]. The treatment starts with recalling the traumatic memory and selecting the most distressing part of this memory with associated dysfunctional thoughts and feelings about oneself. Whilst concentrating on the traumatic memory, the patient is asked to follow the therapists’ fingers with his/her eyes, while being encouraged to follow every association that comes up in the patient’s mind. Repeatedly the patient is asked to report the experiences that come up, which may be cognitive, emotional, somatic, or imaginary. After some sets of eye movements, the patient is asked to report a SUD between 0 and 10, until the disturbance related to the memory reaches a SUD of zero and positive beliefs about oneself related to the trauma are rated as feeling true on a VoC scale of 1–7. Like previous studies [27], we removed stabilization elements (e.g., establishing a “safe place”) from the EMDR standard protocol to avoid interference with the stabilization condition to assure absolute distinction between the two conditions. Like STAIR, the EMDR therapy will be offered twice a week in this study.

All participating therapists hold a post-Master’s psychology degree, have completed STAIR training and advanced training in EMDR therapy, and will provide therapy in both treatment conditions. They will be supervised by experienced trainers who were trained by the originators of both treatment protocols (i.e., Shapiro and Cloitre). Adherence to treatment will be encouraged in several ways. Prior to the start of treatment, a supervised case-conceptualisation will be made in order to plan the treatment sessions. Furthermore, the therapists will be asked to fill out protocol-specific checklists and to explicitly report and explain deviations from the treatment protocol after each session. In addition, all treatment sessions will be recorded on video and a random selection of these recordings will be evaluated during monthly group supervision meetings (one hour for EMDR therapy and one hour for STAIR). Additional supervision will be provided via e-mail and telephone upon request from the therapist. Ultimately, a random selection of individual therapy sessions (33%) will be rated for adherence by two trained psychology graduates using both EMDR and STAIR specific fidelity checklists.

### Procedures

During the regular intake procedure conducted by a clinician not involved in this study and without influence on assignment to the conditions, patients with PTSD due to repeated sexual and/or physical abuse during childhood will be asked to participate in the study. After their approval by signing an informed consent form, they will be assessed on the inclusion and exclusion criteria.

After assessment for eligibility (T0) and baseline assessment (T1; see Fig. 1), the patients will be randomly assigned to one of the two conditions. The randomization will be executed by a computerized randomization programme [28], creating a list of randomized assignment per location (strata). Randomization blocks of six or eight will be employed (divided over four strata based on treatment location) and generated by a study-independent collaborator. The assignments will be put into envelopes per location and will be picked for each patient in order from top to bottom as listed by the computerized randomization. Within each randomly assigned condition, the participants will be assigned to one of the therapists, based on availability. During treatment, assessments will take place after every eight sessions, post-treatment, and at 3 and 6 months follow up.

Due to the nature of the trial, participants and clinicians in the study cannot be blinded to treatment assignment. The research assistants (students studying for a Master’s degree in psychology) who will carry out the assessments will receive intensive training on the interviews and do not have any interest in the outcomes of this study.

### Assessments

#### Assessment of eligibility for inclusion

Eligibility for inclusion of patients will be assessed by using the Clinician-Administered PTSD scale for DSM-5 (CAPS-5) [29, 30]. A symptom is considered present only if the corresponding severity score is rated 2 or higher (on a 0–4 scale) [29]. When a patient scores positive on all the symptoms belonging to PTSD, the diagnosis is established. As part of the CAPS-5, the Life Events Checklist for DSM-5 (LEC-5) [31], will be used to screen and identify repeated or multiple sexual and/or physical abuse before the age of 18 years by a carer or other person in a position of authority. In order to exclude patients with high risk of suicidality, those with a score of 3 on item 9 of the BDI-II [24, 32] will be excluded.

#### Main outcome variable

The main study parameter will be the severity of PTSD symptoms, assessed by the PTSD Symptoms Scale-Self Report [33, 34]. This is a 17-item self-report scale developed as a brief measure of PTSD symptom frequency in trauma victims. Items are scored on a 4-point scale from 0 (“not at all/only one time”) to 3 (“almost always/five or more times a week”). The English version [33] and Dutch version [35] have good psychometric properties.

#### Secondary outcome variables

As a secondary outcome variable, the presence of a PTSD diagnosis will be assessed using the CAPS-5 [29, 30]. The questions in the CAPS-5 about trauma clusters (the sexual and/or physical abuse) will be asked instead of traumatic events, as all the participants were exposed to multiple or repeated trauma. The CAPS-5 will also be used for deciding whether a patient is an early completer or not.

At the moment, a validated interview or questionnaire adhering to the ICD-11 criteria for complex PTSD is not available. We will therefore use the Structured Interview for Disorders of Extreme Stress-Revised (SIDES-R) [36], (Carlier IVE, Jongedijk RA, Lamberts RD, Gersons BPR. Dutch translation of Structured Clinical Interview for DES NOS, unpublished), more specifically the 37-item version developed by Ford et al. [37], as the best available instrument to assess the severity of complex PTSD symptoms. In addition, validated questionnaires measuring the different complex PTSD symptoms will be used. More specifically, to index trauma-related thoughts and beliefs, the Posttraumatic Cognitions Inventory (PTCI) [38] will be used. Patients have to score on a Likert scale from 1 (“I totally disagree”) to 7 (“I totally agree”). Psychometric properties for the English and Dutch [38, 39] version were found to be good. Trait dissociation will be measured using the Dissociative Experiences Scale (DES-II) [40]. The DES-II is a 28-item self-report questionnaire with scores ranging from 0 to 100. Scores above 20 or more conservatively, above 30, suggest pathological dissociation. The DES has been used in well over 200 published studies and its psychometric properties are well-attested [41]. To index interpersonal difficulties, the Inventory of Interpersonal Problems (IIP) will be used [42, 43]. Each of the 32 items of the IIP can be scored on a 5-point scale from 0 (not at all) to 4 (very strongly). The Difficulties in Emotion Regulation Scale (DERS) [44], (Neumann A, Koot H. Nederlandse vertaling van de Difficulties in Emotion Regulation Scale, unpublished) will be used to measure difficulties in emotion regulation. Each item is rated on a 5-point scale and has been validated in clinical [45, 46] and nonclinical populations [44, 47].

The Brief Symptom Inventory [48, 49] will be used to measure symptoms of general psychopathology. This is a self-report instrument measuring the severity of complaints. Each item can be rated on a 5-point scale from 0 (not at all) to 4 (a lot). The reliability, validity, and utility of the BSI have been tested in more than 400 research studies [50, 51]**.**

The Structured Clinical Interview for DSM-IV Axis II personality disorders (SCID-II) [52] will be used to determine the presence of a personality disorder (cluster B and C). This interview is considered as the gold standard in semi-structured assessment instruments for personality disorders. The Dutch translation and adaptation was developed by Weertman, Arntz, and Kerkhofs [53] and validated by Weertman Arntz, Dreessen, Van Velzen, and Vertommen [54].

The EQ-5D will be used to index health-related quality of life [55]. This is a questionnaire consisting of five dimensions with three levels each (from having no problems until being unable to execute an activity). The questionnaire has been validated in different groups and in different countries [56].

Healthcare consumption and productivity loss in patients will be indexed to measure the cost-effectiveness of both treatments, using a case record form based on a form used in other studies [57–59].

Other variables that will be included in the study are age and gender of the participating patients, and socio-economic status, psychotropic medication use at baseline and during treatment, and level of education. After the first session in which psycho-education is given and a case-conceptualisation is made, the expectancy rating of the therapist and the patient will be measured by asking to what extent they expect that the patient will profit from the treatment to which he or she is allocated, using a VAS scale from 0 (not at all) to 100 (totally). Drop-out rate and deterioration of the patient will be registered (i.e., the number of extra face-to-face contacts with a healthcare professional, including additional medication consultations, to avoid crises). Patients who drop out of the study will engage in assessments at every time point as far as possible.

### Power and sample size calculation

To our knowledge, methods for power calculation in the context of linear mixed models are unavailable. A power calculation based on repeated measures analysis of variance (ANOVA) was therefore used as a conservative approximation, with treatment condition as the between-subjects factor and time as the within-subjects factor. For 2 × 5 (between-subjects (treatment condition) × within (pre-treatment, after every eight sessions, post-treatment, 3 month follow up and 6 month follow up) repeated measures ANOVA (alpha 0.05, power 0.80, correlation between measures 0.5, and small effect-size (*f)* 0.10), a total sample size of 122 subjects will be required (*G* power) [60].

### Data analysis

Descriptive analyses will be used to evaluate demographic, clinical and baseline characteristics of both treatment arms. Analyses will be performed on the intent-to-treat basis and the sample of completers: the data of all randomized participants will be analysed using both groups as defined at randomization. On the continuous outcome measures, the groups will be compared using linear mixed models. Baseline scores will be included as covariates, time as a categorical variable, and treatment condition as a fixed effect.

## Discussion

There is debate about the treatment of PTSD related to physical and/or sexual abuse during childhood. A history of (chronic) traumatisation during childhood has been linked to the development of distinct symptoms, such as problems in affect regulation, negative self-concept, and interpersonal problems, which are symptoms that are categorized under the name of complex PTSD [10].

Many experts in the trauma field consider a phase-based approach as the preferred treatment for individuals suffering from the consequences of (repeated) early childhood interpersonal trauma, with symptoms of complex PTSD [8, 12, 61]. Proponents of such a treatment trajectory argue that with the addition of a stabilization phase prior to a trauma-focused treatment, these individuals are more likely to profit from, and less likely to drop out of treatment. Stated differently: “*without stabilization, operating at least at an implicit relational level, no trauma-focused intervention can have a durably positive outcome in the treatment of Complex PTSD*” [62]. Conversely, proponents of the application of immediate evidence-based treatments for this target population argue that a stabilization phase could delay or restrict access to trauma-focused treatments, thereby preventing immediate benefit from those treatments [18]. Because of this controversy, the results of the present study may deliver a pivotal contribution to the field, and the worldwide debate about the treatment of individuals suffering from PTSD due to prolonged or repeated childhood abuse, which may lead to the development of more effective, tailor-made treatments for this group of patients.

An improvement in study design relative to previous studies is that the present study specifically focuses on the added value of stabilization. Assessing the two groups after the same amount of treatment sessions (after 8 or 16 sessions) may provide answers to the question as to which of the two conditions works best and in what phase of treatment. Comparing the two conditions post-treatment makes it possible to answer the question whether STAIR has an added value over EMDR only. As some previous studies found that treatment gains emerged only months after treatment has ended [12], the follow-up assessments are pivotal. To ensure that patients engaging in this study represent a group with severe psychopathology, our inclusion and exclusion criteria state that patients must *repeatedly* be exposed to sexual and/or physical abuse during childhood (i.e., complex trauma). Further, psychotic symptoms and bipolar disorders are not excluded with the only exception of those displaying *acute* suicidality at T0. Finally, to increase the generalizability of the results of the present study, we will also include male patients.

There are some potential limitations of the present study that need to be noted. First, instead of exposure therapy as the trauma-focused components, EMDR will be used, rendering a direct comparison more difficult with previous studies that employed PE as the trauma-focused therapy. However, same as PE, EMDR is one of the first-choice trauma-focused treatments for PTSD [1, 2]. An advantage of using EMDR therapy rather than imaginal exposure is that most therapists in the research setting are already well-educated in using EMDR therapy, as EMDR maybe more widely used than other trauma-focused therapies, at least in Europe [63–65].

Furthermore, the quality of assessment of symptoms of complex PTSD is not ideal. While in clinical practice, the term complex PTSD is widely used, definitions of complex PTSD have changed over time and validated diagnostic interviews are not yet available for the current construct of CPTSD as proposed for ICD-11 [11]. Therefore, in this study, the SIDES is used to index severity of complex PTSD and additionally, a variety of psychometrically sound questionnaires to measure the different symptoms associated with complex PTSD separately (e.g. interpersonal functioning, emotion regulation, dissociation).

Most importantly, the results of this study might help to ameliorate treatment for patients suffering from PTSD due to repeated sexual and/or physical abuse during childhood thereby providing important insights on how to improve tailor-made guideline recommendations for this target group.

## Trial status

The second protocol version was finished in May 2016. Trial enrolment started on 5 September 2017 and recruitment is ongoing as of 31 December 2018 Additional file 1.

## Additional file

Additional file 1: SPIRIT 2013 checklist: recommended items to address in a clinical trial protocol and related documents. (PDF 132 kb)

## Abbreviations

ANOVA: Analysis of variance
BDI-II: Beck Depression Inventory
BSI: Brief Symptom Inventory
CAPS: Clinician-Administered PTSD Scale for DSM-5
DERS: Difficulties in Emotion Regulation Scale
DES-II: Dissociative Experiences Scale
DSM-5: Diagnostic and Statistical Manual of Mental Disorders
EMDR: Eye movement desensitization and reprocessing
EQ-5D: Euroqol-5D
ICD-11: International Classification of Diseases 11
IIP: Inventory of Interpersonal Problems
LEC-5: Life Events Checklist for DSM-5
PSS-SR: PTSD Symptoms Scale-Self Report
PTCI: Posttraumatic Cognitions Inventory
PTSD: Posttraumatic stress disorder
SCID-II: Structured Clinical Interview for DSM-IV Axis II
SIDES-R: Structured Interview for Disorders of Extreme Stress-Revised
STAIR: Skills training in affect and interpersonal regulation
SUD: Subjective unit of distress
VAS scale: Visual analogue scale
VoC: Validity of cognition
